# P02.13. Improving stress and resilience for military healthcare providers: results from a pilot study

**DOI:** 10.1186/1472-6882-12-S1-P69

**Published:** 2012-06-12

**Authors:** M Bingham, A Inman, J Walter, W Zhang, W Peacock

**Affiliations:** 1 Samueli Institute, Alexandria, USA; 2 Brooke Army Medical Center, Fort Sam Houston, San Antonio, USA

## Purpose

Healthcare providers (HCP) who care for traumatically injured service members are at risk for stress, burnout, and decreased clinical effectiveness. After two decades of war, the intensive work environment and stress associated with caring for these service members and their families are damaging the emotional and physical well-being of our military HCPs. This pilot study explored the effects of an Integrative Restoration (iRest®) intervention in HCPs at a large military medical center.

## Methods

Volunteers from a military medical center were asked to participate in an intervention developed and tailored for military HCPs. Participants were asked to attend 6 one-hour weekly sessions (offered 2 times/week) and practice at home once a week. Outcome measures included stress, sleep disturbance, resilience, burnout, compassion and satisfaction. Goals were to assess interest, feasibility, and logistics of the intervention and measure stress and resilience (pre-post intervention). Using a mixed methods approach, self-report instruments, home practice diaries, and written/oral feedback were collected to measure acceptability and satisfaction.

## Results

Overall, 14 participants completed the study (74%). The majority was female (80%), nursing providers (85%). Perceived stress decreased significantly from pre to post intervention (p=.0005) and also secondary traumatic stress (p < .01). Although resilience, sleep, and burnout measures did not change significantly, they all trended in the desired directions, a promising sign for a small pilot study. Qualitative findings from diaries and feedback will also be presented.

## Conclusion

The study demonstrates an accepted and effective intervention for military HCPs with significant changes in stress levels, despite a small sample size. Given the ever-increasing demands placed on military HCPs, evidence-based interventions contributing to the resilience of these staff warrant further attention. Results of this study informed a 3-year funded RCT with military couples. Status and details of this new study will also be discussed; recruitment began in June 2011.

